# Supplementary material for: Recovery of metagenomic data from the Aedes aegypti microbiome using a reproducible snakemake pipeline: MINUUR
Source: Wellcome Open Res. 2023 May 26;8:131. Originally published 2023 Mar 23. [Version 2] doi: 10.12688/wellcomeopenres.19155.2 (PMC10412942; doi:10.12688/wellcomeopenres.19155.2)
Supplement: Peer review history

# Peer review: Reviewer response for version 2

**DOI:** 10.21956/wellcomeopenres.21596.r64469

**Published:** 2023-08-09

**Authors:** Mariana Rocha David

**Affiliations:** 1 Laboratório de Mosquitos Transmissores de Hematozoários, Instituto Oswaldo Cruz, Fundação Oswaldo Cruz (FIOCRUZ), Rio de Janeiro, Brazil

The present manuscript, "Recovery of metagenomic data from the *Aedes aegypti* microbiome using a reproducible snakemake pipeline: MINUUR" is a method article that presents a workflow to analyse non-host reads from existing genomic data with the aim to extract host associated microbe sequences. Using 62 *Aedes aegypti *samples, the results indicated that it is possible to identify bacterial phyla and genera usually found in association with this mosquito species. There was a remarkable variation in taxonomic diversity between sequencing projects, as well as there was some variation between samples (which is in line with mosquito microbiota studies). This workflow can increase the knowledge about bacteria potentially associated to *Aedes aegypti*, although it is not possible to distinguish between symbionts and contaminants (as pointed by the authors). In the following text I present some questions and suggestions about the present manuscript.

 Methods:

- Do the authors also consider searching for fungal, protozoan, and viral DNA sequences?

Results:

- Figure 6C: it seems to be an empty plot.

Discussion:

- What is the meaning of “constituent” in, “Whilst the nature of the samples can act as confounder given differences in sample handling preparation, we note the relative abundance of the key phyla, and constituent genera, are varied across these projects”?
- It is also important to consider that some bacteria could be on the surface of the mosquito, since the samples were not prepared for microbiota analysis (e.g. they did not have their external surface sterilized with ethanol).

Is the rationale for developing the new method (or application) clearly explained?

Yes

Is the description of the method technically sound?

Yes

Are the conclusions about the method and its performance adequately supported by the findings presented in the article?

Yes

If any results are presented, are all the source data underlying the results available to ensure full reproducibility?

Yes

Are sufficient details provided to allow replication of the method development and its use by others?

Yes

Reviewer Expertise:

Vector ecology, symbiotic interactions, vector competence, arbovirus transmission

I confirm that I have read this submission and believe that I have an appropriate level of expertise to confirm that it is of an acceptable scientific standard.

# Peer review: Reviewer response for version 2

**DOI:** 10.21956/wellcomeopenres.21596.r57419

**Published:** 2023-05-31

**Authors:** Ellen Cameron

**Affiliations:** 1 European Molecular Biology Laboratory, European Bioinformatics Institute (EMBL-EBI), Wellcome Trust Genome Campus, Hinxton, UK; 2 Wellcome Sanger Institute, Hinxton, England, UK

In “Recovery of metagenomic data from the *Aedes aegypti* microbiome using a reproducible snakemake pipeline: MINUUR [version 2]”, Foo et al. describe a new Snakemake workflow, *MINUUR*. *MINUUR* is then used to characterize the *Aedes aegypti *microbiome in publicly available whole genome sequencing datasets on the European Nucleotide Archive. This workflow functions at the interface between whole genome sequencing and metagenomic sequencing to recover metagenomic assembled genomes (MAGs) of bacterial symbionts and contaminants in a host ( *Aedes aegypti*) through the implementation of commonly used metagenomic tools.

 Overall, *MINUUR* is rooted on the basis of re-assembly of non-mapped reads which will allow for the successful recovery of present symbionts. Numerous tools exist for metagenomic assembly and binning and currently, *MINUUR* utilizes *MEGAHIT* for assembly and *metaBAT2* for binning. On this front, I believe that there are two opportunities for further development of the workflow:

**Assembly**: Is there a reason why authors chose to use *MEGAHIT *and did authors also evaluate *metaSPAdes* as an assembler? *metaSPAdes* has been known to give better quality assemblies but may have higher resource requirements/run time. It would be nice for the workflow to also give users the option of using *metaSPAdes*.

**Binning**: Currently, the workflow only implements *metaBAT2* for binning. Including additional binners (e.g., *CONCOCT*) and a bin refinement tool (e.g., *dasTOOL*, *metaWRAP* bin refinement module, *BinSPreader, GraphBin*) may improve the recovery of metagenomic assembled genomes. I would recommend including adding in additional binning/bin-refinement to have more robust evaluation of assemblies.

 It is evident that the authors have put a lot of time and effort into the development of a user-friendly Snakemake workflow. The GitHub is well organized and the Snakemake workflow structure (e.g., Snakefile, rules directory, scripts, environments) is very clean. I also appreciate their inclusion of test data which will allow for users to easily test out the workflow. The authors have also prepared detailed documentation for set-up and operation of the workflow in a Jupyter notebook. I have a few minor suggestions for the workflow which I think may make it more accessible and user friendly:

**More automation in setup requirements**: In the documentation, clear instructions are provided for users on what databases are required for operation. However, users are required to download these databases manually. Including the ability for databases to automatically download would make setup and operation more user-friendly. This could be done through downloading from the hosts of the databases (e.g., *wget* to link in a Snakemake rule with a “Download databases” binary True/False option in the config). Additionally, on the topic of automation in the workflow setup, the authors provide great instructions on how users can use *MINUUR *for alternative hosts by creating the bwa index as outlined in their Jupyter notebook. I believe that this could also be included as a simple option in the config as an additional optional rule in the workflow to make the tool more user-friendly and to simplify the application of this workflow in non-mosquito hosts.

**Conda environments**: In the instructions for *Running MINUUR*, users are instructed to use the flag *–use-conda* with the snakemake command. I noticed in the GitHub that there are currently six conda environments required for execution. Having numerous conda environments will increase the time required during the initial setup of the workflow, but also can take up a lot of storage. If there are not version conflicts, it would be great if the total number of conda environments could be reduced by including more of the dependencies in a single environment. Alternatively, I also noticed that there is a Dockerfile but no mention of this – will there be the option to run the workflow using docker/singularity in the future instead?  

 The authors demonstrated the utility of their workflow by applying it to numerous publicly available datasets and presented these key findings. I have a few minor comments about their figures that I believe will help to address overall readability:

**X-axis labels: **I noticed in several of the figures that the x-axis labels were often difficult to read because there was minimal spacing between adjacent labels. I would adjust this to ensure the labels can be clearly read. Specific instances where I noticed this include: Figure 3B, Figure 4B, Figure 4C, Figure 6A.

**Axis Inconsistencies: **Within some of the figures subfigures, there are inconsistencies in the axis labelling/limits. While this does not directly interfere with readability and interpretation, updating these will just make for much higher quality and cohesive visuals. Specific examples: i) Figure 3 – subfigure A and B do not expand to 0 but C does. Figure B includes (%) in the y-axis title, but figure C includes % after each number in the y-axis tick labels.

**Figure 3B: **I am not clear on what data is being presented here. Based on the text where this figure is referenced, I assume it is the number of samples that each genus has been identified in (e.g., a taxon that was identified in 31 samples == 50%?) but the figure legend and axis labels do not clearly communicate this. Perhaps including “detection” somewhere on the graph/figure legend text would help to clarify this?

**Figure 3C: **On initial look at this graph, I was confused because it looked like your bars were not adding up to 100%. However, on closer inspection, I realized that this is due to the light shade being plotted for “Other” and that it just has the appearance of being ‘white’/empty space. I think that either including an outline or choosing a darker shade in place of the pale grey would improve this. I would also italicize the genera names in the legends.

**Figure 4: **I would include the colours for the MAG classification as a legend next to the graphs to increase readability. I think it could also be interesting to plot the genome sizes and N50 scores for the low-quality MAGs (perhaps as a supplementary figure?).

**Figure 5A: **Faceting the panels by the MAG quality instead could be a nice alternative to allow for cross-project comparisons? This is just personal preference though depending on what you are trying to highlight here.

**Figure 6C**: I cannot see any data plotted here and only am able to see the empty plots (E.g., axis labels, facet labels). This is likely just due to an issue with the geom style not cooperating when inserted as a picture in Word (I commonly see this with geom_point() and inserting as a PDF image in Microsoft Word).

 I believe that the inclusion of a eukaryotic scoring also increases robustness of the workflow to not only focus on prokaryotic MAGs. However, I am interested as to how “eukaryotic contamination” is defined. Is this serving to identify instances where host ( *i.e., Aedes aegypti*) reads have made it into the newly assembled reads or could this instead be the presence of eukaryotic symbionts and/or be indicative of blood meal diet source? Clarifying this in the text will help to contextualize the usage of the BUSCO scoring.

 I believe that the authors should also include a table in the supplementary data highlighting the MAG quality information (completeness, contamination) including what sample and what project these MAGs were identified in. For example, authors make mention of a specific instance of PRJNA385349 not containing any high-quality MAGs and I would be interested to see what the quality of MAGs were ( *i.e.*, were they nowhere close to being high-quality or were they on the threshold?).

Is the rationale for developing the new method (or application) clearly explained?

Yes

Is the description of the method technically sound?

Yes

Are the conclusions about the method and its performance adequately supported by the findings presented in the article?

Yes

If any results are presented, are all the source data underlying the results available to ensure full reproducibility?

Yes

Are sufficient details provided to allow replication of the method development and its use by others?

Yes

Reviewer Expertise:

Metagenomics, bioinformatics, symbiotic interactions

I confirm that I have read this submission and believe that I have an appropriate level of expertise to confirm that it is of an acceptable scientific standard, however I have significant reservations, as outlined above.

# Peer review: Reviewer response for version 1

**DOI:** 10.21956/wellcomeopenres.21234.r56050

**Published:** 2023-04-17

**Authors:** Taylor Reiter

**Affiliations:** 1 Arcadia Sciences, Berkeley, California, USA

In, “Recovery of metagenomic data from the *Aedes aegypti* microbiome using a reproducible snakemake pipeline: MINUUR,” Foo *et al.* describe a snakemake workflow to leverage contamination in whole genome sequencing data from mosquitos to discover microbiome constituents. Contamination is rampant in WGS projects, so this workflow cleverly leverages that information to inform new biological insights.

 The steps the authors chose for their workflow make sense. I have alternative preferences for some steps — using fastp instead of FastQC → cutadapt → FastQC and using sourmash gather for taxonomic annotation of reads instead of kraken2. However, none of these choices are terribly consequentially important to the downstream outcomes. I think the tool could do a better job of assessing residual host contamination in unmapped reads and in resultant MAGs. This is particularly concerning as most high- and medium-quality MAGs were larger than expected. Some strategies I can think of to do this would be:

1. Mapping against a pangenome instead of a reference genome in the initial step to remove more reads.
2. Using bbduk to remove contamination with a low k-mer size (k=21 or k=17).
3. Assessing for eukaryote or host-specific genes in the unmapped reads (e.g. using DIAMOND or something similar) or in the assemblies (using BUSCO or something similar).

I also find it unfortunate that that authors chose not to include GTDBtk as part of the workflow, as most people would probably like to run a similar step.

 Another concern I have with this approach is detecting true host contamination (e.g. symbionts) vs. process-oriented contamination (kit contamination, index hopping, etc). It would be interesting to compare the kraken2 or GTDB-tk labels against lists of organisms that are frequently contaminants. If the authors could also back track and determine the concentration of DNA used in sequencing and compare this to the number of organisms detected, this might highlight whether process-oriented contamination drives results in any samples. The authors may also look into GUNC as another tool for assessing contamination separate from checkM.

 I have concerns around the implementation of the MINUUR workflow. As a data analysis project, the process the authors went through to run this analysis is phenomenally documented and I found it easy to follow. However, the authors present the workflow as a tool that others could use for other WGS projects. I think in this context, the workflow lacks some adherence to software engineering principles that could greatly facilitate downstream use:

1. Some of the software tools that are installed via conda by the workflow do not have recorded versions (humann, samtools, bam2fastq to name a few).
2. Some of the R packages that are used are not installed via conda, which will make it difficult for new users to use the pipeline.
3. The workflow lacks tests. I don’t think third party tools need unit tests, but the workflow could be greatly improved by including unit tests for the new R and python scripts that are introduced there in.
4. It appears that users must download databases themselves, which creates cognitive burden associated with getting the workflow up and running.
5. It would be super useful to have a small toy dataset that will quickly run through the whole pipeline so that users could validate that the workflow is up and running on their system.

Some smaller things I highlighted while reading include:

1. In the first paragraph of the results section, it would be helpful if you could report the percent of unmapped reads instead of absolute numbers.
2. It would be interesting to compare the kraken2 results against the GTDB tk results.
3. Some of the font in the figures is very small, making it difficult to read.

Is the rationale for developing the new method (or application) clearly explained?

Yes

Is the description of the method technically sound?

Yes

Are the conclusions about the method and its performance adequately supported by the findings presented in the article?

Partly

If any results are presented, are all the source data underlying the results available to ensure full reproducibility?

Yes

Are sufficient details provided to allow replication of the method development and its use by others?

Partly

Reviewer Expertise:

Metagenomics, automation with workflows, contamination detection, bioinformatics of sequencing data

I confirm that I have read this submission and believe that I have an appropriate level of expertise to confirm that it is of an acceptable scientific standard, however I have significant reservations, as outlined above.

# Response

**Published:** 2023-05-18

**Authors:** Eva Heinz

**Affiliations:** Liverpool School of Tropical Medicine, UK

Reviewer Responses  

**I think the tool could do a better job of assessing residual host contamination in unmapped reads and in resultant MAGs. This is particularly concerning as most high- and medium-quality MAGs were larger than expected. Some strategies I can think of to do this would be:**  

1. **Mapping against a pangenome instead of a reference genome in the initial step to remove more reads **
2. **Using bbduk to remove contamination with a low k-mer size (k=21 or k=17).**
3. **Assessing for eukaryote or host-specific genes in the unmapped reads (e.g. using DIAMOND or something similar) or in the assemblies (using BUSCO or something similar).**

We thank the reviewer for these suggestions. We have added BUSCO to the pipeline as a step to assess the final assemblies. The step can either be used to look for Eukaryotic (--auto-lineage-euk) or prokaryotic marker genes (--auto-lineage-prok). To address the reviewers query, we used BUSCO to search for eukaryotic specific genes within high and medium-quality MAGs. The manuscript has been updated with the following sections:  

- Within the methods section we added the following sentence to account for the inclusion of BUSCO: *“In addition, BUSCO (v5.4.7) can be included to search for eukaryotic or prokaryotic specific genes in the final MAGs as an additional quality assurance metric”*
- And in the results, we include the following section: *“We further applied BUSCO to assess eukaryotic contamination in the final assemblies. Eukaryotic contamination was 3.24% (0 – 4.47%) and 2.26% (0.4 = 4.7%) on average in high and medium quality MAGs respectively (Supplementary Figure 2,* *Extended data* *)”*  

This graph is now included in Supplementary Figure 2 (under the new doi https://doi.org/10.5281/zenodo.7941078): **Supplementary Figure 2: BUSCO (v5.4.7) Assessment of Eukaryotic Contamination**. BUSCO percentage of complete eukaryotic marker genes from the eukaryota_odb10 database. Y-axis represents the percentage of genes from the eukaryota_osb10 database present in the assembly, X-axis represents the sample and bin id (sample.bin).  

**I also find it unfortunate that that authors chose not to include GTDBtk as part of the workflow, as most people would probably like to run a similar step.**  

 This is a fair comment, however, we decided to exclude GTDB-Tk from the workflow since another large database dependency (The Genome Taxonomy Database) would be problematic. Also, since MAGs are not always acquired using this pipeline, setting up GTDB-Tk and downloading the required reference databases as part of the workflow would be computationally and time expensive, with no potential return. We decided it would be better for individuals who wanted to repeat this analysis to run GTDB separately if they were able to acquire MAGs from their host of interest using the workflow.  

**Another concern I have with this approach is detecting true host contamination (e.g. symbionts) vs. process-oriented contamination (kit contamination, index hopping, etc). It would be interesting to compare the kraken2 or GTDB-tk labels against lists of organisms that are frequently contaminants. If the authors could also back track and determine the concentration of DNA used in sequencing and compare this to the number of organisms detected, this might highlight whether process-oriented contamination drives results in any samples. The authors may also look into GUNC as another tool for assessing contamination separate from checkM.**

 We thank the reviewer for highlighting this. We agree and have addressed this limitation in text in the final paragraph of the discussion, with citations of key papers discussing the issue. Within the discussion, we have added the following paragraph:  

 “A limitation of this workflow is knowing whether this data is indicative of symbionts associated to the *Aedes aegypti *microbiome, or sequencing contamination (78–80). We believe the majority of our results support the identification of true microbial symbionts to the mosquito microbiome; our taxonomic classifications from MAGs and KRAKEN2 read assignments are congruent with previous studies (4,10,13,18,19,25,72,74). However, we also identify likely contaminants such as *Brevidensovirus *(Figure 3) *, *which have previously been identified as ZIKV stock contamination (81). Discerning between symbiont or contaminant from results generated through this workflow requires further cross reference to the host-microbiome literature in question. Further analysis can also help answer questions of contamination or symbiont results, such as measuring species genetic similarity with other sequenced host-symbionts.”  

**I have concerns around the implementation of the MINUUR workflow. As a data analysis project, the process the authors went through to run this analysis is phenomenally documented and I found it easy to follow. However, the authors present the workflow as a tool that others could use for other WGS projects. I think in this context, the workflow lacks some adherence to software engineering principles that could greatly facilitate downstream use**  

1. **Some of the software tools that are installed via conda by the workflow do not have recorded versions (humann, samtools, bam2fastq to name a few). **
  We have updated all of these packages accordingly – we thank the reviewer for pointing this out. (https://github.com/aidanfoo96/MINUUR/tree/main/workflow/envs)
2. **Some of the R packages that are used are not installed via conda, which will make it difficult for new users to use the pipeline.**
  The R packages have been added with specified versions (https://github.com/aidanfoo96/MINUUR/tree/main/workflow/envs)  
3. **The workflow lacks tests. I don’t think third party tools need unit tests, but the workflow could be greatly improved by including unit tests for the new R and python scripts that are introduced there in.**
  We have added a unit test for the pipeline which tests all third party and R packages are can be properly configured within their conda environments specified in the pipeline. Github actions re-runs this with each push request. We have also added the GitHub actions badge to the Readme to make users aware of the workflow’s status.
4. **It appears that users must download databases themselves, which creates cognitive burden associated with getting the workflow up and running.** 
  We certainly agree with this point. However, we decided for a long-term perspective that users should download the respective databases themselves. The first reasoning for this was that the teams who host these databases do a fantastic job maintaining the databases in dedicated repositories – this is something we are unable to do. Second, these databases are updated often to reflect the growing number of reference data available; this is something we do not have the resources to do. Instructions for installing and configuring these databases is well documented, and repositories are hosted (such as Ben Langmead’s repository cited in the paper) to provide precompiled databases that a user can download and implement. 
5. **It would be super useful to have a small toy dataset that will quickly run through the whole pipeline so that users could validate that the workflow is up and running on their system**.
  This is a great suggestion by the reviewer – we have added a toy dataset into the data repository of the pipeline (https://github.com/aidanfoo96/MINUUR/tree/main/workflow/data) and configured the sample_list and sample_table to automatically point to this dummy data. We have also added instructions to the JupyterBooks page so that when a user initializes the pipeline with the required databases they can run through the workflow with the dummy data.
6. **In the first paragraph of the results section, it would be helpful if you could report the percent of unmapped reads instead of absolute numbers**  
  We have changed the following section.
7. **Extraction of Non-Reference Reads Post-Alignment to *Aedes aegypti*, **now reads: *“91.9% of reads aligned to the AaegL5.3 reference genome, while 4.6% of reads did not map to the AaegL5.3 reference genome”*.  
8. **It would be interesting to compare the KRAKEN2 results against the GTDB-Tk results**  
  Thank you to the reviewer for the suggestion – we included in the results section of the original manuscript a summary of the KRAKEN2 classifications and GTDB-Tk classifications. Within the section **“Relationship between KRAKEN2 Taxonomic Classifications and MAG Recovery”** we included the following sentence: *“taxonomic* *classifications with a high number of KRAKEN2 assigned reads (>100,000) are akin to the taxonomic classifications of medium and high-quality MAGs (Supplementary Figure 3).”*  
  We have expanded on this in the updated version of the manuscript with the following: “ *For example, across multiple samples, Serratia, Elizabethkingia and Wolbachia were assigned >100,000 reads and resulted in six, 27 and 13 medium and high-quality MAGs respectively.”*  
9. **Some of the font in the figures is very small, making it difficult to read.**
  We have examined our figures and made the bar charts in Figure 4 larger to better distinguish the x-axis labels.
